# The Effect of Frankel’s Stabilization Exercises and Stabilometric Platform in the Balance in Elderly Patients: A Randomized Clinical Trial

**DOI:** 10.3390/medicina55090583

**Published:** 2019-09-11

**Authors:** Grzegorz Mańko, Magdalena Pieniążek, Sabina Tim, Małgorzata Jekiełek

**Affiliations:** 1 Department of Ergonomics and Physiological Effort, Institute of Physiotherapy, Jagiellonian University Collegium Medicum, 31-531 Krakow, Poland; g.manko@uj.edu.pl; 2 Institute of Physiotherapy, Faculty of Health Sciences, Jagiellonian University-Collegium Medicum, 31-126 Krakow, Poland; pieniazek.m@interia.pl; 3 Student Scientific Group Institute of Physiotherapy Faculty of Health Sciences Jagiellonian University-Collegium Medicum, 31-126 Krakow, Poland; sab.thim@gmail.com

**Keywords:** elderly patients, rehabilitation, risk of fall, exercise programs, functional recovery

## Abstract

*Background and Objectives:* Every year, older people are becoming a larger part of the population. In a couple of years medicine is going to struggle with specific disorders and their consequences, where one of them are falls. Fall prevention involves a use of strengthening exercises, equivalent exercises, stabilometric platforms, and special exercise programs. Almost the entire brain is involved in maintaining correct balance. Reduction of a volume of gray matter negatively affects balance. Single exercise sessions do not significantly improve balance. In order to achieve satisfactory results at least 10 training sessions are required. The aim of this study is to determine if there is a correlation between a risk of falls, gender, and a risk of falls and the age of the subjects. Another reason to conduct that research was to assess the effectiveness of Frankel’s exercises and training of using the stabilometric platform in rehabilitation, which aims to reduce the risk of falls among elderly people. *Materials and Methods:* The study involved 40 elderly patients referred for physiotherapy to a rehabilitation center. The patients were divided into two groups of 20 people. In experimental group 1 (C) Frankel’s stabilization exercises were used; in experimental group 2 (E) a stabilometric platform was used. The correlation between the risk of falls and age as well a risk of falling and the gender of the examined persons was taken into consideration. The effect of therapy that uses stabilization exercises and the stabilometric platform on the risk of falls in the examined persons was assessed using the Tinetti scale. Clinical control was performed using the Tinetti scale, before and after a two-week rehabilitation period. *Results:* The study showed no correlation between the degree of risk of falling and age, and between the risk of falling by the elderly and gender. There were also changes in the results obtained by patients after using the training, both with the use of Frankel’s stabilization exercises as well as with the use of the stabilometric platform. Patients using the dynamometric platform obtained higher results in the Tinetti test after treatment. *Conclusions:* In the examined sample, no correlation was found between the risk of falls and age as well as the risk of falls and gender. Both Frankel’s exercises and training with the use of the stabilometric platform were effective in a rehabilitation program aimed at reducing the risk of falls among the elderly.

## 1. Introduction

Due to advances in medicine, life expectancy has increased. The Central Statistical Office forecasts predict that in Poland people aged 65 and over will constitute an increasing percentage of the population [1]. Currently, the percentage share of older people in the total population in Poland is about 18%, while in 2050 it may increase to 32.7% [2]. Elderly people are exposed to specific, chronic disorders leading to disability and reduced quality of life, called great geriatric problems. One of these problems is falls [3]. The risk of falls increases with age. Falls are the cause of injuries, fractures, and complications associated with damage on body structures. This leads to immobilization and fear of falling again, making older people less willing to take up activities [4]. Women and people over the age of 80 are more likely to fall [5]. The increasing risk of falls is caused, among others, by multi-morbidity, multi-medication, blurred vision, hearing, muscle weakness, osteoporosis, or changes in body posture [6]. For the body to properly maintain static and dynamic balance, it must have an efficient sensory system that receives stimuli, a central nervous system that processes and interprets stimuli, and a neuromuscular system that responds appropriately to information received from the CNS [7]. Research suggests that almost every area of the brain is responsible for balance. However, the cerebellum, basal ganglia, thalamus, hippocampus, and frontal and parietal lobes have the greatest role here. The cerebellum is responsible for motor coordination and movement planning. Reducing the gray matter of the cerebellum negatively affects balance. Recently, an increasing role has been attributed to the hippocampus. It transfers information to long-term memory but also remembers spatial information. The reduction of gray matter within the basal and thalamic nuclei, placed in the brain that control motor functions, negatively affects balance. Similar conclusions were made when examining the volume of gray matter of the parietal lobe; reducing this volume worsens balance [8]. Although falls are most common among older people, they are not the only group where they can occur. Falls often occur in people with Parkinson’s disease and multiple sclerosis, and after strokes, wherever brain tissue degeneration occurs [9]. Physical activity is crucial in preventing falls [10]. Equivalent strengthening and gait exercises bring the most benefits [11]. We have many methods of exercises aimed to prevent falls of the elderly. The OTAGO (Program to prevent falls) Tai Chi exercise set and the use of modern game consoles, where biofeedback elements are used, are becoming increasingly popular [3]. Biofeedback is an effective technique that responds to errors arised during movement onongoing basis [12]. Biofeedback is an objective tool for monitoring the progress of rehabilitation [13]. The use of virtual reality is an interesting method of supporting classic kinesitherapy [14,15]. However, individual trainings on the platform or classic kinesitherapy do not bring long-term effects. To improve balance, one must attend at least 10 exercise sessions [14].

The aims of the study are to assess the relationship between the risk of falls and age and gender, and to assess the effectiveness of two rehabilitation methods to reduce the risk of falls in the elderly.

## 2. Materials and Methods

The study was a randomized clinical trial. The group’s representativeness was ensured by randomizing patients to treatment groups. From the group of patients referred to a rehabilitation center on the basis of a clinical referral, in order to improve motor function, persons meeting the criteria for inclusion in the study were selected. Then a group of 40 people was drawn from among them, who underwent therapy using balance exercises according to Frenkel or the stabilometric platform. All studies were conducted with the consent of the patients, ensuring anonymity. Before starting the therapy, the questionnaires of patients qualified for the study were coded as follows: C or E (experimental group 1 or experimental group 2). Respectively, patient’s initials were numbered from 1 to 20 so the person who was responsible for the statistical analysis could not have identified the patient by using the data in the questionnaire. The study was conducted in the period from April to July 2016. Patients were informed to which of the study groups they were qualified, how the therapy would be carried out, what research tools would be used during the therapy, and what the purpose of the rehabilitation proceedings was. The project was positively examined by the Bioethics Committee (Opinion No. 122.6120.342.2016 dated 28 April 2017). A positive opinion was also received from the Research Team of the Krzeszowice Movement Rehabilitation Center (Opinion No. 2/2017 of 29 March 2017).

### 2.1. Participants

The study included a selected group of patients, aged 76–84, undergoing a two-week rehabilitation stay at the Krzeszowice Movement Rehabilitation Center. The respondents were divided by means of a randomization program into two groups so that the groups were comparable in terms of age and gender. In the experimental group 1 group (C) there were 11 men and 9 women, while the experimental group 2 (E) consisted of 12 men and 8 women. Table 1 presents the median and interquartile range (IQR) of age and body mass index (BMI) in groups C and E.

Criteria for inclusion in the study:

- at least 75 years old,
- expressed written consent to participate in the study, undergoing therapy using exercises according to Frankel’s or stabilometric platform, undergoing Tinetti evaluation before and after therapy;
- physical condition enabling movement and self-service in everyday activities assessed by a primary care physician referring to a rehabilitation stay on the basis of medical history and medical examination;
- mental state ensuring cooperation during the performance of tests, assessed by primary care physician referring to a rehabilitation stay based on an interview and a medical examination;
- medical referral for physiotherapy.

The criteria for inclusion of the patient in the study were additionally assessed by the doctor and physiotherapist when admitting the patient to the rehabilitation center in order to verify and assign him/her to the appropriate rehabilitation group. The assessment was conducted by an interview in terms of exclusion criteria from the study on mental diseases and an interview with the up and go test to verify the inclusion criteria in terms of physical condition. The up and go test is based on the patient getting up from the chair at a command, overcoming a quiet pace of 3 m, turning 180 degrees, returning to the chair, and then sitting down. Patients who were unable to perform the test did not meet the physical condition criterion for mobility and were excluded from the study.

Exclusion criteria for the test:

- age over 85 years;
- cancer;
- mental disorders and behavior caused by the use of psychoactive substances, affective disorders, schizophrenia, dementia (Parkinson’s disease, Alzheimer’s disease, dementia);
- severe comorbidities: stroke, cervical myelopathy;
- previous operations related to knee or hip joint replacement;
- people with unstable coronary artery disease and unstable blood pressure;
- period of exacerbation of cardiological and rheumatic diseases;
- inflammatory bowel disease.

Patients with mental illness and patients who were unable to move independently and were not able to perform self-service activities were not referred to the Rehabilitation Center in which examinations were performed. Despite this, in order to maintain the high quality of the examination, patients were evaluated by a physician and a physiotherapist by an interview.

### 2.2. Instruments

For all 40 patients qualified for the study and undergoing therapy, the clinical examination was performed by using the Tinetti scale, which allows one to assess the balance in situations related to physical activity during which falls most often occur. As a result of the test, a maximum of 28 points could be obtained (16 in the balance assessment section and 12 in the gait assessment section). Achieving below 19 points indicates a high risk of falling, a score between 19 and 23 points means the occurrence of a tendency to fall and reaching above 23 points means low risk of falls.

### 2.3. Interventions

The experimental group 1 used balance exercises according to Frenkel’s exercises, which included learning how to walk together with upper limbs, and learning how to turn around, sit down, and get up by using various everyday objects such as chairs, beds or traces painted on the floor. This is a standard exercise program used in a rehabilitation center where tests are performed. Patients performed the following exercises:

1. The patient lay on his/her back, alternately flexed and straightened his/her leg in the hip and knee joint.
2. The patient lay on his/her back, at the same time abducted and put on straight legs.
3. The patient lay on his/her back, performed bending movements of the knee and hip joint alternately in the air, and imitated the pedaling movements of a bicycle.
4. The patient lay on his/her back, bent and extended his/her knee and hip joint with one limb, and abducted and adducted with the other limb.
5. In a sitting position on a chair with bent knees, the patient alternately bent the lower limb in the hip joint.
6. In a sitting position on a chair with knees straightened, the patient alternately bent the lower limb in the hip joint.
7. In a sitting position on a chair with bent knees, the patient alternately marked circles or patterns with feet on the floor.
8. From a sitting position on a chair with knees bent, the patient got up from the chair.
9. In the sitting position on the chair, the therapist gently nudged the patient to unbalance.
10. In the standing position, the therapist gently nudged the patient to disturb the balance.
11. The patient in a standing position rotated left and right without lifting his/her feet off the ground.
12. The patient in a standing position rotated 360 degrees with the movement of the feet.
13. The patient put his/her foot on the stair and then placed the other foot next to the one standing on the stair.
14. The patient put his/her feet in designated places.

The patients performed exercises every day for 15 min during a two-week long stay, which included 10 training sessions. For the people in the experimental group, the Zebris stabilometric platform was used along with the factory-available training plan. The software included with the platform is an interactive game for training proprioception and balance. It involves steering the box to reflect visible falling tomatoes on the monitor, so that they reach the table. The control is done by balancing the body in the frontal plane, through variable pressure on the left and right foot. Similar to the control group, the training session was daily performed during the rehabilitation stay and lasted 10 min. The patient clinical control was performed by using the Tinetti test twice, before training sessions and after the end of the rehabilitation period (the day after the last training session), to compare the results.

### 2.4. Data Analysis

In order to describe the variables used in the study, the central tendency indicator, which is the median, and the measure of dispersion, which is IQR, were used. Spearman rank correlation (or Spearman rank correlation, Spearman rho) was used for statistical analysis to check for the existence of a correlation between the variables studied. The Wilcoxon test was used to check the significance of the differences obtained before and after training within groups C and E, and the Mann–Whitney U test to check the significance of the difference between the improvement of the result of patients in groups C and E. The age of patients was also distinguished between below and above 80 years of age to half-divide patients who, according to inclusion criteria, should be between 75 and 85 years old.

## 3. Results

In the conducted statistical analysis, no correlation was found between the risk of falls and age and gender. In addition, there was a difference in the results of patients in groups C and E on the Tinetti scale after the end of therapy.

From the data obtained during the study, it was noticed that the risk of falling in the studied group of people was high, because as much as 87.5% of people had a score of 0–18 points on the Tinetti scale. Other respondents exhibited an average risk of falling. None of the examined persons obtained a score above 23 points before training. Table 2 presents the distribution of the risk of falls in patients participating in the study.

Table 3 shows the distribution of activities in which the patients were unable to obtain the maximum number of points before starting therapy. The activities that caused the biggest problems for the patients before training were 360 degree rotation, sitting, walking path at 30 m, and maintaining the stability of the torso while walking. In these categories, all patients did not obtain the maximum number of points. Activities that did not cause problems for most patients were balance during sitting, where all patients obtained the maximum number of points, getting up from a place, where only 15% of patients did not reach maximum, and initiating gait immediately after the command, where only 8% of patients also did not reach maximum.

Analyzing the gender distribution in the study group before training, 76.5% of women showed a high risk of falling, while the remaining showed an average risk of falling. For men, 95.7% of men had a high all risk score and only 4.3% had a medium fall risk. Table 4 shows the distribution of risk of falls in men and women before therapy.

In terms of age before training 84.62% of patients under 80 years of age showed a high risk of falling, while the remaining 38% of patients had a medium risk of falling. For patients over 80 years of age, 92.86% of patient show a high risk of falling, other patients are at medium risk of falling. Table 5 shows a comparison of the pre-workout fall risk distribution in patients under and over 80 years of age.

In the balance test, the median was 10 points out of 16 possible. The minimum score obtained in this part of the test was 7 points and the maximum was 12 points. In the part related to gait, the median of points obtained was 7 points out of 12 possible. The minimum score obtained was 8 points and the maximum 12 points. Table 6 shows the distribution of points obtained by patients in individual parts of the Tinetti test before training.

An analysis was conducted on the correlation between the risk of falls in patients with age of patients undergoing therapy. There was no statistically significant correlation between the gender of patients and the degree of risk of falling. Similarly, in the relationship between age and risk level, no statistically significant correlation was found. Table 7 shows the results of the Spearman correlation coefficient analysis to examine the existence of correlations between the variables studied.

After using group C training, the activities that caused the most problems to the subjects were sitting, maintaining the stability of the torso while walking, and following the designated walking path. A minimum of 80% of patients in this group did not achieve the maximum result. In group E, as in group C, no improvement in 360-degree turnover was achieved, where in both groups all patients did not achieve the maximum number of points. Table 8 shows the distribution of activities in which the patients were unable to obtain the maximum number of points before starting therapy.

Table 9 presents the distribution of risk of falls in groups C and E after the therapy methods used. After the end of the two-week rehabilitation stay, patients were tested again using the Tinetti scale and subjected to statistical analysis. When assessing the risk of falls in the control group, 90% of patients showed an average risk of falling, while in the experimental group all patients were at low risk of falling.

Table 10 presents the results of the chi-square test for the distribution of the degree of risk of falling after training in groups C and E.

After the end of the therapy, the correlation between the risk of falling and gender as well as the risk of falling and age was analyzed again. No statistically significant correlation was found between patient gender and the risk of falling. Similarly, in the relationship between age and risk level, no statistically significant correlation was found. Table 11 shows the results of the analysis of Spearman’s correlation coefficient to examine the existence of correlations between the variables tested after training.

Analyzing the gender distribution in both examined groups, it was noticed that the majority of women and men after the therapy showed an average risk of falls in the C group. In the E group all women and men obtained results classifying them to a group at low risk of falling. Table 12 contains the results of the analysis related to the distribution of the degree of risk after training in both experimental groups by gender.

In the C group, after the therapy was used among patients below and above 80 years of age, patients with a moderate risk of falling prevailed; among patients in the E group all patients regardless of age obtained results classifying them in the group of low risk of falling. Table 13 presents the distribution of risk of falls in patients in the C and E groups, taking into account the age of the subjects.

Table 14 presents the distribution of points obtained by patients of groups C and E in individual parts of the Tinetti test after training. In group C, the median points obtained by patients in the balance section was 12 points, while in group E it was 15 points. In the gait section, the median was nine points in group C and 12 points in group E.

In order to compare the effectiveness of therapy using Frankel’s exercises and the Zebris stabilometric platform, the differences between the results obtained after the therapy were compared to the results obtained before the start of treatment. In the part of the Tinetti balance test, the median obtained by patients in group C was three points while in group E it was five points. In the gait section, the median improvement in group C was two points and in group E, five points. Considering the Tinetti test as a whole, in group C the median improvement was five points and in group E it was 10 points. Table 15 shows the distribution of the difference in points obtained by patients in individual parts of the Tinetti test after training in relation to the number of points obtained before training.

Table 16 presents the results of the Wilcoxon test, in which the statistical significance of therapy effectiveness was checked in both group C and E. It was shown that in both cases patients obtained more points after the end of training programs compared to the results obtained before the start of the rehabilitation period.

Using the non-parametric Mann–Whitney U test, a statistically significant difference was found between the results in the Tinetti test obtained by group C and group E after the end of training programs. Table 17 shows the results of the Mann–Whitney U test.

## 4. Discussion

The aim of our work was to examine the relationship between the risk of falls and age and gender, and to evaluate the effectiveness of Frankel’s exercises and training on the stabilometric platform to reduce the risk of falls in the elderly. This research confirms the effectiveness of both methods. Comparing the results obtained in the Tinetti test by patients of both groups before and after therapy, their improvement can be seen. However, better results were obtained by group E, performing training on the platform, obtaining a median of improving results throughout the test by 10 points. Group C, where Frankel’s exercises were used, obtained a median improvement of five more points throughout the test. In addition, no correlation was found between age and fall risk, and gender and fall risk. Similar studies were performed by Shahrbanian et al. in a group of women. They confirmed the effectiveness of stabilization exercises and exercises on the platform. However, as in our research, greater improvement in balance was noticed in the group that performed exercises on the platform, using elements of biofeedback [16]. Exercises on the platform are used in virtually every field of rehabilitation, which confirms their effectiveness [3,17]. Exercises on the platform improve the symmetry of loading of the lower limbs, which improved gait and reduced the risk of falling [18,19]. Exercises on the platform are also a more interesting form of rehabilitation than classic exercises, because patients are more motivated; they exercise more willingly by observing their results [17,18]. On the other hand, studies by Kołcz-Trzęsicka et al. suggest that platform exercises should not, however, be used as the only form of therapy, but as a complement to it [1]. The study showed that the risk of falling before and after training does not depend on gender and age, and that there are no significant differences between women and men in the tested functions of the Tinetti test. Weobserved that for both men and women pre-workout clearly had a high risk of falling. However, after training, the proportions between medium and low risk are very similar for both men and women.

Different results of the impact of gender and age on the risk of falling were obtained by Ostrowska et al. She conducted the study using the Tinetti test on a group of 58 residents of a nursing home who were aged 65–93. Based on the analysis, it was noted that age, chronic disease states, lower limb disability, vision disorders, and the use of many drugs are factors that increase the risk of falls. It was also shown that disorders of balance and movement increase with age, while women are more affected by this process [20]. Francis and Rubenstein also indicate female gender as a risk factor for balance disorders [21,22].

Confirmation of the beneficial effects of appropriate exercises can also be found in Żak’s research, which showed that stabilization exercises conducted after 45 min for 12 weeks three times a week, combined with learning how to recover after a fall and learning how to safely change position, improved functional performance and gait in 62.5% of respondents, which in turn reduced the risk of falling [23]. Equivalent exercises on gymnastic balls were also used by Rogers et al. who showed that therapy has a positive effect on static and dynamic balance, and thus reduces the risk of falling [24]. Żak also noted in his other studies that the rehabilitation program used, which used, among others, sensorimotor exercises, facilitating—by stabilizing posture, loading distal parts of the body, and improving concentration—mastering the ability to safely change position and rise after falling, was beneficial for the subjects [25]. In the review of the research carried out by Costello and Edelstein, it was shown that exercises strengthening the muscular strength in combination with equivalent exercises reduce the risk of falls [17]. Research by Vafaeenasab et al. showed that Frankel’s exercises improved both static and dynamic balance among people over 60 years of age [26]. Our studies also confirmed the effectiveness of these exercises.

In planning therapy, it is important to evaluate the difficulty and choose an exercise program so that it is interesting for the patient to receive, then he/she does not lose motivation to continue training [3]. Our research has shown that Frankel’s stabilization exercises and platform exercises improve balance; patients improved in the Tinetti test. It follows that both methods can be used successfully to prevent falls. The most important is the regularity of the exercises performed [19].

## 5. Conclusions

The study showed no correlation between the risk of falls in the elderly and age, and between the risk of falls and gender. This trend was maintained both before and after training with the use of Frankel’s stability exercises and the stabilometric platform. In addition, both methods of therapy have been shown to be effective in reducing the risk of falling in older people, except for the 360 degree rotation component, where both methods of therapy used have proved ineffective. People who underwent therapy using the Zebris stabilometric platform obtained better results in the Tinetti test after the therapy.

## Figures and Tables

**Table 1:** Age distribution and BMI in experimental group 1 (C) and experimental group 2 (E).

Variable	C Group		E Group	
	Median	IQR	Median	IQR
age	79	3.75	78	3.75
BMI	25.01	1.83	25.40	2.08

IQR—interquartile range.

**Table 2:** Distribution of the risk of falling before training in the examined group of people.

Degree of Risk of Falling	Before Training	
	*n*	%
High (0–18 points)	35	87.5
Average (19–23 points)	5	12.5
Low (>23 points)	0	0

**Table 3:** The distribution of activities from the Tinetti test in which patients did not score the maximum number of points.

Activities	Before Training	
	*n*	%
Sitting balance	0	0
Rises from chair	6	15
Attempts to rise from chair	19	48
Immediate standing balance (first 5 s)	24	60
Standing balance	30	75
Nudged (subject at max position with feet as close together as possible, examiner pushes lightly on subject’s sternum with palm of hand 3 times)	32	80
Eyes closed (at max position—see #6 above)	31	78
Turning 360 degrees	40	100
Sitting down	37	93
Initiation of gait (immediately after told to “go”)	3	8
Step length and height	23	58
Step symmetry	28	70
Step continuity	19	48
Path (estimated in relation to floor tiles, 12-inch diameter; observe excursion of 1 foot over about 10 feet of the course)	40	100
Trunk	40	100
Walking stance	34	85

**Table 4:** Distribution of the risk of falling before training in the examined group of people by gender.

Degree of Risk of Falling	Before Training			
	Woman		Man	
	*n*	%	*n*	%
High (<19 points)	13	76.5	22	95.7
Average (19–23 points)	4	23.5	1	4.3

**Table 5:** Distribution of the risk of falling before training in the examined group of people by age.

Degree of Risk of Falling	Before Training			
	Age < 80 Years		Age > 80 Years	
	*n*	%	*n*	%
High (<19 points)	22	84.62	13	92.86
Average (19–23 points)	4	15.38	1	7.14

**Table 6:** Distribution of points obtained in two parts of the Tinetti test before training.

Tinetti Test	Points			
	Median	IQR	Min	Max
Tinetti assessment tool: balance	10	1.75	7	12
Tinetti assessment tool: gait	7	1	8	12

**Table 7:** Spearman’s correlation coefficients between the risk of falling and gender, and age before training.

Statistics		*p*	r
**Spearman correlation coefficient**	Correlation: risk of falling and gender	0.073	−0.287
	Correlation: risk of falling and age	0.855	−0.030

**Table 8:** The distribution of activities from the Tinettia test in which patients did not score the maximum number of points.

Activities	After Training			
	Group C		Group E	
	*n*	%	*n*	%
Sitting balance	0	0	0	0
Rises from chair	0	0	0	0
Attempts to rise from chair	2	10	2	10
Immediate standing balance (first 5 s)	4	20	0	0
Standing balance	7	35	2	10
Nudged (subject at max position with feet as close together as possible, examiner pushes lightly on subject’s sternum with palm of hand 3 times)	7	35	2	10
Eyes closed (at max position—see #6 above)	7	35	0	0
Turning 360 degrees	40	100	40	100
Sitting down	17	85	3	15
Initiation of gait (immediately after told to “go”)	0	0	0	0
Step length and height	0	0	0	0
Step symmetry	5	25	1	5
Step continuity	2	10	0	0
Path (estimated in relation to floor tiles, 12-inch diameter; observe excursion of 1 foot over about 10 feet of the course)	16	80	1	5
Trunk	17	85	1	5
Walking stance	14	70	0	0

**Table 9:** Distribution of the degree of risk of falling after training in groups C and E.

Degree of Risk of Falling	After Training			
	Control Group		Experimental Group	
	*n*	%	*n*	%
Average (19–23 points)	18	90	0	0
Low (>23 points)	2	10	20	100

**Table 10:** Chi-square test for distribution of risk of falling in groups C and E after training.

Statistics	χ^2^	df	*p*
Chi-square test	29.57	1	0.000

**Table 11:** Results of Spearman’s correlation coefficient analysis regarding the risk of falling by gender and by age after training.

Statistics		*p*	r
**Spearman correlation coefficient**	Correlation: risk of falling and gender	0.888	−0.023
	Correlation: risk of falling and age	0.605	0.084

**Table 12:** Distribution of the risk of falling after training in groups C and E by gender.

Degree of Risk of Falling	After Training							
	C Group				E Group			
	Woman		Man		Woman		Man	
	*n*	%	*n*	%	*n*	%	*n*	%
Average (19–23 points)	8	88.89	10	90.91	0	0	0	0
Low (>23 points)	1	11.11	1	0.09	8	100	12	100

**Table 13:** Distribution of the risk of falling after training in groups C and E by age.

Degree of Risk of Falling	After Training							
	C Group				E Group			
	Age < 80 Years		Age > 80 Years		Age < 80 Years		Age > 80 Years	
	*n*	%	*n*	%	*n*	%	*n*	%
Average (19–23 points)	12	92.31	6	85.71	0	0	0	0
Low (>23 points)	1	7.69	1	14.29	13	100	7	100

**Table 14:** Distribution of points obtained in two parts of the Tinetti test after training.

Tinetti Test	Points							
	C Group				E Group			
	Median	IQR	Min	Max	Median	IQR	Min	Max
Tinetti assessment tool: balance	12	1	9	14	15	1	13	15
Tinetti assessment tool: gait	9	0.75	8	11	12	0	11	12

**Table 15:** Distribution of point difference obtained before and after training in the Tinetti test.

Test Tinetti	Difference of Points Obtained by Patients After Training							
	Control Group				Experimental Group			
	Median	IQR	Minimum	Maximum	Median	IQR	Minimum	Maximum
Tinetti assessment tool: balance	3	2	1	5	5	2	2	7
Tinetti assessment tool: gait	2	1.75	0	5	5	1.75	3	8
Total	5	2	3	12	10	2	7	12

**Table 16:** Test results of the Wilcoxon test statistical significance of point difference within the group before and after training.

Statistics	Group C		Group E	
Wilcoxon Test	Z	*p*	Z	*p*
Tinetti assessment tool: balance	−3.94	0.000	−3.96	0.000
Tinetti assessment tool: gait	−3.77	0.000	−3.96	0.000
Tinetti test	−3.94	0.000	−3.42	0.000

**Table 17:** Mann–Whitney U test results compared to the average improvement in the results obtained by control and experimental patients in the Tinetti test.

Statistics	*p*	Mann–Whitney U Test
Tinetti assessment tool: balance	0.000	344.50
Tinetti assessment tool: gait	0.000	368.00
Total	0.000	351.500
